# Developing a model for prevention of malnutrition among children under 5 years old

**DOI:** 10.1186/s12913-020-05567-x

**Published:** 2020-08-05

**Authors:** Mohammad Mohseni, Aidin Aryankhesal

**Affiliations:** 1 School of Health Management and Information Sciences, Iran University of Medical Sciences, Tehran, Iran; 2 Health Management and Economics Research Center, Iran University of Medical Sciences, Tehran, Iran; 3 Department of Health Services Management, School of Health Management and Information Sciences, Iran University of Medical Sciences, Tehran, Iran

**Keywords:** Prevention, Pattern, Malnutrition, Under 5 years old children

## Abstract

**Background:**

Serious consequences leading to the increase of infectious diseases and mortality of children justifies the importance of interventions for eradication of malnutrition. Thus, this study aimed to provide a model for the prevention of malnutrition among children under 5 years old (CU5) in Iran.

**Methods:**

This paper is part of a bigger study, conducted in 2017 using a mixed methods approach. A model for prevention of malnutrition in CU5 was proposed based on the earlier steps, with the cooperation and consultation of experts and specialists. In the final step, a Delphi method was used to determine the validity of the proposed model.

**Results:**

The main dimensions of the prevention model of malnutrition for CU5 in Iran included four level: basic causes, interventions, outcomes and impact. The proposed interventions are presented based on twelve areas: structural, intersectoral, political, economic, sanitary, health-oriented, research, educational/cultural, evaluation related, production, infrastructures and legal. Based on these areas, 118 solutions were finally selected for the final model. This model is designed based on the current conditions in different regions of Iran, the factors related to child malnutrition, affective context on policy making, the content of previous policies, the process of policy making in Iran, and key stakeholders and actors in policy making.

**Conclusion:**

In order to prevent malnutrition, the causing factors should be identified and resolved. The adopted policies should be, more seriously, based on the presence of key stakeholders and actors. Most of the existing nutritional problems among children are because of inappropriate consumerism culture and habits in families and its transfer to children.

## Background

Early years of childhood, as the most important years of life, can affect the health status of whole life seriously [1, 2]. The first 1000 days of life are so momentous that low and inadequate nutrition in this period may lead to an irreversible growth decrease along with cognitive ability disorders and performance reduction [3–6]. Growth monitoring during childhood is one of the most important health care means of children, and growth disorder is the first recognizable sign of medical, social, and especially nutritional problems [7].

Malnutrition is a condition in which a deficiency, excess or imbalance of energy, protein and other nutrients occurs [8]. Approximately half of all children under five (CU5) mortalities are attributable to under nutrition. Under nutrition exposures children to a higher risk of dying from common infections and slower recovery when infected [9]. Malnutrition among children also affects their cognitive-sensory function and consequently disrupts their ability to have a productive and efficient life [10].

This type of malnutrition is caused by many factors such as inadequate care in pregnancy period, low literacy of family members, inadequate community malfunctions (injustice, war, natural disasters, etc.), polluted environments, poor nutrition of the household, frequent and severe infections, inadequate supply of food, and poverty, especially [11]. Despite the importance of malnutrition and its relevance to the cause of important diseases, this issue continues to be underestimated [12] .

Considering the fact that stunning, underweight and wasting are among the most important indicators of malnutrition, the current study aimed to use these indicators to investigate malnutrition among children. In 2018, globally 149 million CU5 (22%) were stunted [13] and 49.48 (7.3%) million wasted [14]. The prevalence of underweight, wasting and stunning in Iran, according to the latest national study were 3.8, 4 and 4.6%, respectively [15]. Although malnutrition prevalence has been declining in recent years, Iran is still far from a World Without Malnutrition [9].

Using the indigenous models, specific for each region of world, needs to be considered to reduce malnutrition and improve the situation. Hence, given the importance of malnutrition in children, the aim of this study was to develop a model for the prevention of malnutrition among CU5 in Iran.

## Methods

**Fig. 1:**
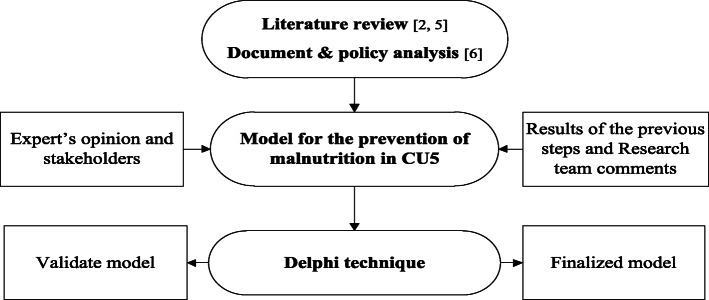
Research steps. The final model is the result of a mixed methods approach in four step. In the final step, a Delphi method was used to determine the validity of the proposed model

This paper is a part of a bigger study that was conducted in 2017 using a mixed methods (quantitative and qualitative) approach in four steps (Fig. 1). The first and second steps were a systematic review and a policy analysis conducted and published earlier [2, 5, 6]. In the third step of the study, based on the findings of the first two steps, a malnutrition prevention model was developed for the CU5, enjoying the cooperation and consultation of experts and specialists. In the final step of the study, a Delphi method was used to determine the validity of the model. The Delphi technique is an approach for gathering data used to gain consensus among respondents within their domain of expertise. This is achieved through a series of rounds using questionnaires (Table 1), where information is fed back to panel members [16, 17].

**Table 1:** Delphi Form

Dimensions and Policy Options	For developing a model for prevention of malnutrition among children under 5 years old, what do you think about the following options?				
Structural	Strongly Disagree	Disagree	No-Idea	Agree	Strongly Agree
1.					
2.					

The initially proposed model was formulated in the form of a questionnaire. For this step, the study sample consisted of 38 key policy makers, senior health system managers, specialists and faculty members of nutrition departments of universities. The inclusion criteria were as follows: the individuals with education and research history on nutrition, especially CU5, senior managers with the history of conducting research (at least 5 years), individuals with the experience of executive jobs (at least 5 years), and finally, having effective experience related with policy making in this area. Snowballing method was also used to detect the eligible samples for entering the study.

Data collection tool comprised of three sections: demographic information of respondents, policy options, and also a section for recording the participants’ responses about the proposed options. The validity of the questionnaire was evaluated through the opinions of experts in this field. To assess the reliability, a pilot study was done on five individuals. A summary of the objectives along with a proposed model was sent to the experts in two rounds. The results of the first phase were analyzed and a revised model was developed based on the comments. It was then sent back to the experts and to collect their final comments. To prepare the final model, the Delphi step findings were analyzed by descriptive method. If the agreement was over 75%, the policy option was approved and if the agreement was between 50 and 75%, the policy option entered the next round. Moreover, the agreement below 50% would lead to the rejection of options [18]. At the end, the final model was formulated based on the approved dimensions and aspects.

## Results

**Fig. 2:**
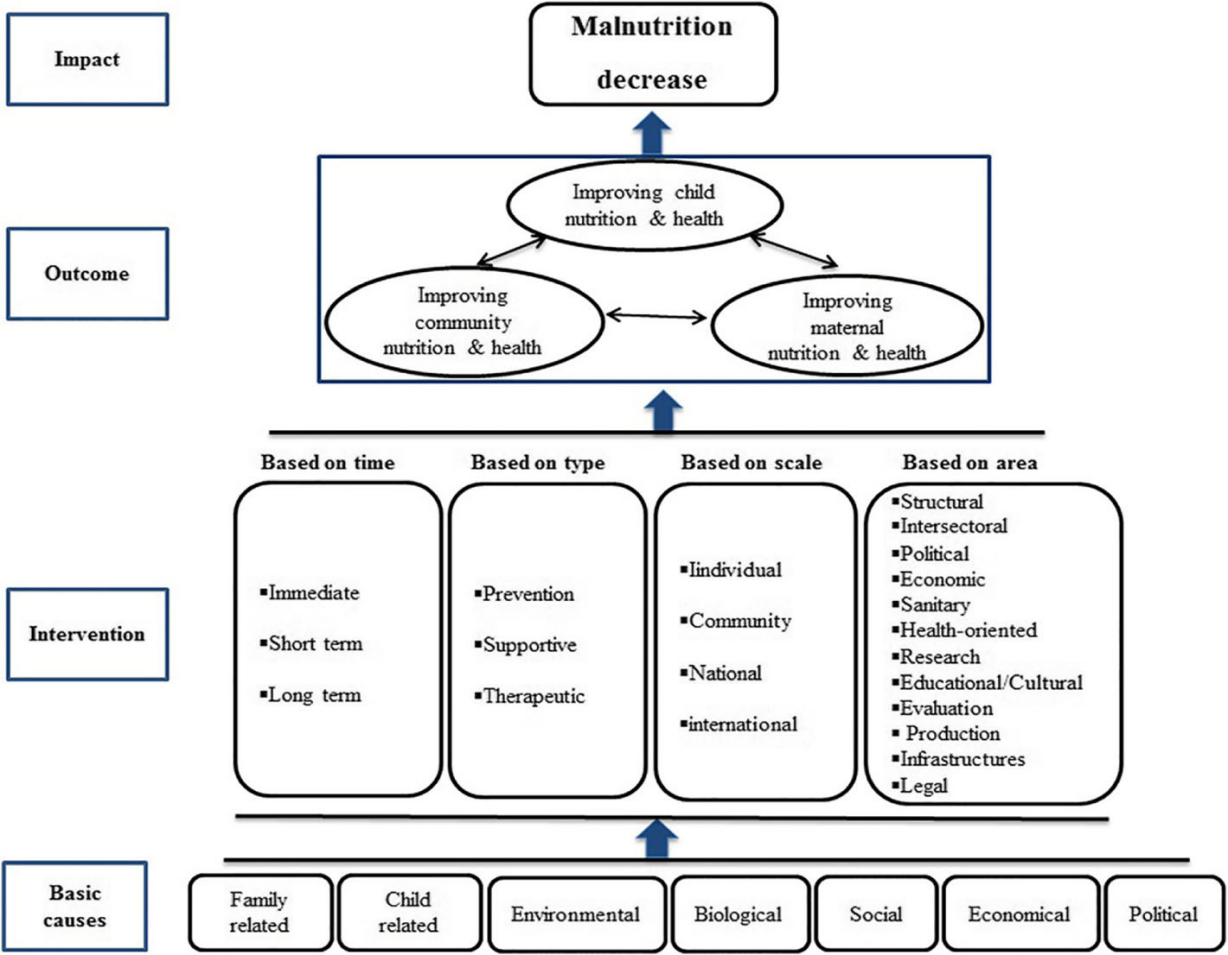
The main dimensions of model for prevention of malnutrition among children under 5 years old. The main dimensions of the final model included four level: basic causes, interventions, outcomes and impact. The interventions can implement based on the area, scale, type, or time to reduce the children malnutrition

In order to design the primary model (Fig. 2), the findings of the first phase of the study [5], the factors related to malnutrition in Iranian CU5, were classified into six categories: the social, economic, biological, environmental, child-related and family-related causes. The findings of the meta-analysis study [2] showed that the prevalence of malnutrition was higher in the deprived regions of the country. Therefore, different regions of the country need different interventions; some regions need urgent and short-term interventions while some preventive and supportive interventions, due to their low economic situation and higher prevalence compared with the standards assigned by World Health Organization. These issues are considered in the present model. Given the cultural, ethnic and regional differences, the level of education and different food styles in different regions of the country, we need individual and social policies which are included in the final model. The findings from the interviews [6] are also used in more details for the regions in need of intervention.

The first and second phases of the Delphi attended by 25 and 20 individuals respectively. In Table 2, the proposed interventions are presented within the following twelve areas: structural, intersectoral, political, economic, sanitary, health-oriented, research, educational/cultural, evaluation related, production, infrastructures and legal. Based on these domains, 118 policy options were finally selected for preventing malnutrition among CU5.

**Table 2:** Policy options of the final model for malnutrition prevention among children under 5 years old in Iran

Dimensions	Policy Options
Structural	Strengthening the activates of the Supreme Council for health and Children Nutrition through structural reform
	Making the ministry of health to operate the leadership completely as a policymaker and chief responsible for the children nutrition
	Strengthening the staff structure to improve the community nutrition at the health deputy of Health Ministry
	Strengthening the line structure in executive agencies of the cities, such as health networks and nutrition experts for more coordination
	Strengthening the capacity of the PHC system to solve the nutritional problems of mothers and children
	Establishing the community-based structures in the processes of food security level promotion at the Ministry of Health
	Increasing the public participation in implementing operational programs through participation in the executive body
	Using more expert personnel in the field of production, processing and food health
Intersectoral	More coordination for nutrition-related policies in beneficiary organizations
	Using the graduates of management and policy making in the field of nutrition
	Increasing the linkages between research centers and the institutions of policy making and decision making
	Strengthening the effective participation of the scientific community to implement the programs of children nutrition
	Strengthening the effective participation of private sector to implement the programs of children nutrition
	Making the related organizations such as TV, Ministry of Culture and NGOs more sensitive to the topic of children nutrition
	Implementing the approved health plans with more commitment by beneficiary organizations
Political	Increasing the share of food and nutrition in macro policy makings of the health sector
	Activating the committees of supreme council for health more to run the existing laws better
	Paying more attention to evidence-based policy making on policies of children nutrition in related organizations
	Decentralized regional policy making for more adaptation to the different regions of the country
	Proper implementation of some nutrition laws that are not properly implemented, such as National Document of Nutrition and Food Security
	Formulating the comprehensive planning for production, consumption and demand with the participation of all organizations involved in this field
	Prohibiting radio, television and other mass media from advertising heath threatening food products
	Putting taxes on health threatening food products
Economic	Absorbing the financial resources completely by the organizations, which have a credit for the children nutrition.
	Receiving more financial resources from international organizations which are active in the field of nutrition and poverty
	Reforming the laws and programs related social justice to balance the distribution of per capita income in the country and the improve the poor condition
	Identifying the groups under the poverty line complete and being committed to support their nutrition fully
	Employment and income generation for unemployed, young, low-income and poor people through formal plans
	Making food subsidies purposeful based on differences in economic conditions of different regions
	Making production subsidies purposeful and applying as well as modifying the price tools
	Increasing the credit of per capita for a hot meal in rural kindergartens
	Supporting micro-industries, particularly in agriculture and food to increase rural finance capacity
	Supporting rural development projects to improve the economic conditions of the village and its residents
	Providing facilities and subsidies to private sector to invest in producing healthy food products
	Providing a milk subsidy appropriate with desired food basket to increase the per capita consumption of milk and its products
	Financial support for educational programs, and advertisements related to health and nutrition of mother and child health in TV
Sanitary	Reducing agricultural crops by monitoring the responsible organizations and continuous evaluation of products
	Removing unauthorized additives in the processing and maintaining of food
	Supporting the projects of food safety promotion at industrial units
	Paying special attention to the elimination of health problems in deprived areas with insecure food
	Teaching families about the principles of food hygiene in providing and maintaining child food
	Teaching families on hygiene (hand wash, sanitary waste and ...)
	Teaching families on parasitic diseases and their transfer and cure
	اIncreasing public health measures to prevent intestinal parasitic infection among children
	Training to reduce the consumption of unhealthy and low value food by children through media, health and educational centers
Health oriented	More access to health care, especially in areas with poor economic status
	Complete children health care, especially in the first 1000 days of life
	The possibility of children’s access to foods full of micronutrient in all regions of the country
	Facilitating the access to fresh and natural food, especially fruits and vegetables for mothers and children
	Identification of nutritious and proper native food to feed children and develop the culture of its supply and consumption
	Emphasis on the proper various dietary for mother and the child and to receive the nutrients they need
	Complete implementation of breastfeeding programs, especially during the first six months
	Complete implementation of complementary nutrition programs after the first six months
	Supporting the promotion of breastfeeding and proper supplemental nutrition with appropriate training and incentives.
	Preventing from the reduction of the quantity and quality of the food basket due to poverty, inflation and food prices
	Preparation of desired food baskets in accordance with culture, tradition and the status of different regions
	Providing comprehensive nutrition program to deal with micronutrients deficiency, especially Vitamin A, D, Iron, and Zinc
	Changing the composition of supply pattern to reduce the consumption of salt, oil and sugar
	Strengthening family planning and proper birth spacing
	Focus on mother’s nutrition prior to pregnancy
	The mother and child nutritional treatment system at the Ministry of Health, especially in hospitals and private clinics
	Establishing and strengthening of the nutritional counseling units in health centers
	Establishing and strengthening of intensive care unit for children with severe malnutrition in all hospitals
	Supportive measures to the cure the children having diseases due to malnutrition
	Encouraging and supporting the production and consumption of nutritional supplements among susceptible and sick children and mothers
	Strengthening the children’s growth monitoring program at community health centers
	Strengthening the supplemental programs of micro-nutrition for children emphasizing on family education
Research	Development of research capacities and interdisciplinary specialties in universities
	Studying on applied researches related to children’s nutrition problems
	Studying on enriching major nutrient with a variety of micronutrients more powerful
	Research studies on the causes of malnutrition in different regions of the country
	Use of research findings from other countries to address the causes and problems of child nutrition
Educational/Cultural	Acceleration of absolute illiteracy reduction, especially in deprived regions faster
	More use of new promotional tools such as social networks for proper advertisements and training on health related topics
	Promoting the nutritional culture and literacy through the huge potential of social media
	Proper implementation of nutritional labeling system to aware consumers
	Increasing the knowledge and skills of general practitioners as the first level of nutrition care in the community
	Training and retraining of health workers in the field of family proper nutrition, especially mother and child
	Training and retraining of health care staff, especially nurses in pregnancy and pediatrics departments
	Teaching mothers and girls, especially high school girls, about mother and child nutrition
	University educations on proper food and nutrition based on community needs
	Cultivation to limit the production and consumption of low-value and harmful food
	Cultivation and promotion of using vegetables and fruits based on the desired food basket
	Use of cultural and traditional tools of communities to educate and promote the nutritional literacy
	Presenting food and nutrition education for personnel (at the beginning, during or work retraining)
	Including food and nutrition in different steps of reviewing and editing textbooks
	Public education about the nutritional value of milk and its products to increase its per capita consumption
	Planning and implementing the comprehensive nutritional education program for mothers
	Informing parents about nutritional, supplement and labeling standards which are proper for the children
	Enhancing the empowerment and participation of people in implementing the programs related to removing children malnutrition and appropriate nutrition
Evaluation	Exact evaluation of the nutrition activities for children and presenting report to the supreme Council for Health
	Launching the monitoring system of micronutrient and the nutritional status for children and pregnant mothers in health centers
	Continuous monitoring of food and nutrition status through electronic registration systems
	Continuous monitoring of educational and promotional food related to nutrition programs, especially in the mass media
	Strengthening the monitoring and control system on the import of food and raw materials in the food industry
	Strengthening the monitoring system of diseases caused by food among children
	Update and standardize the growth monitoring system to measure the anthropometric status of children
Production	Paying attention to the production of food products which support the health of children by producers and supervisory organizations
	Paying more attention to quality rather than only the quantity in agricultural and food products
	Reducing food waste based on general education and culture making
	Formulating and revising the standards of food, raw materials and packaging standards
	Reducing the production costs and food prices
	Supporting the development of service centers in the agriculture and food industries
	Supporting the business development projects in small-scale food industries by the Ministry of Industry
	Reducing the excessive use of chemical pesticides and supporting the biological methods to control pests
	Promotion of planting vegetables and consuming them, especially in rural regions
	Organizing and optimizing the traditional production units of milk and its products
	Supporting the production of organic foods
	Supporting the production of probiotic foods
infrastructures	Development of infrastructures such as health network, transportation and communications to have access to more food, health services and health facilities.
	Technology developing in the specialty fields of food and nutrition and adaptation to advanced technologies
	Promoting the level of technology in the food chain, from production to consumption
	Strengthening and equipping food labs in provincial health centers to control the quality of food products
Legal	Providing the comprehensive national document for nutrition in the country
	Enforcing the observatory laws on the quality and health of foods
	Development and continuation of the national program for the enriching the flour with iron and folic acid
	Complete implementation of the laws which prohibit the promotion of harmful food
	Developing and enforcing the laws which protect of high-quality food producers

## Discussion

The prevalence of malnutrition among Iranian CU5 is much lower than the global average and is in relatively good condition. About contextual causes, mother education level, father education level, child gender, birth weight, and age group were mentioned as the most important factors in the literature.

Based on evidence, the main factors in malnutrition are low socioeconomic status of family, parental education level, household health index, health literacy, nutrition culture, maternal characteristics such as BMI, nutrition during pregnancy, and the number of childbirths, child characteristics such as age, gender, birth weight and common infectious diseases [19–25]. Considering that nutritional problems are multi-factorial, different creating causes need to be resolved to prevent them [26]. The problem of food insecurity or malnutrition in a region would be solved only if all its contextual, and mediating and immediate factors are addressed [27].

The interventions are suggested to be classify based on the area, scale, type, or time to reduce the children malnutrition. Considering the contextual causes, the most important intervention could be maternal measures. Maternal interventions, particularly during pregnancy, may have intergenerational effects and on birth weight and child growth [28]. Other interventions in the studies include providing nutritional aids and paying cash, micronutrient supplements such as vitamin A, fertility and children health (immunization, prenatal care, the presence of a skilled person at childbirth, and the treatment of childhood common diseases), hand washing program, supplement foods enriched with micronutrient [29–33]. Another powerful potential strategy to prevent malnutrition in poor and sick children is consuming nutritional supplements [34]. Studies have also shown that the prevalence of malnutrition in lower social classes is higher [35] and by improving the economic status and reducing poverty, the chance of a better nutrition at higher income levels is higher. In this regard, some studies have shown a relationship between income and a reduction in the risk of stunting [36–39].

Low cost and time are the two points mentioned in previous studies regarding the implementation of interventions. Results of the study by Peru et al. showed that nutritional interventions are low cost and may improve growth in the short or midterm [40]. Clombati et al. also showed that in countries with a high prevalence of malnutrition, low cost and short-term interventions are easily applicable and effective [41]. Furthermore, Shimpton et al. showed that these interventions should start from the early pregnancy or at birth [42].

Nevertheless, results of some studies showed that some interventions had low impact. The results of three meta-analysis studies showed that the use of micronutrient as an intervention was effective in improving the development of children, but iron and vitamin supplements did not have a significant effect in the improvement of children’s growth [43]. A study by Bandari et al. in rural areas of India revealed that educational interventions aimed at increasing energy consumption and improving nutritional methods for infants from 6 to 18 months could not improve their weight [44]. Some double-blind controlled studies also showed that zinc intake could not improve the growth of malnourished children [45].

In general, it seems that screening in short intervals, the status of children can be monitored regularly and the effectiveness of interventions can be controlled in order to modify them. It should also be noted that health policies, including nutrition, differ greatly from other policies, and interests and objectives of the governors have a direct impact on nutrition policies [46]. Some studies showed that strong political support programs were of the main strengths of nutritional policies; and such support could improve the nutritional status of the children [47, 48].

The findings of policy analysis in the current study showed that using evidence, documents, policies, previous experiences, and experiences of other countries, a successful model for preventing child malnutrition can be developed for the country. A model that, in addition to the children’s health and nutrition needs, involves all individuals, organizations and stakeholders, considers the health of mother and child before the childbirth and has the ability to align the activities of all stakeholders. Furthermore, a nationwide program and a comprehensive document in the field of the nutrition for CU5 is needed; to do so, strengthening of the political process is crucial [49].

Some studies presented a model for preventing and reducing malnutrition in children. Using appropriate models to improve the quality of services in different organizations can be considered to develop the final model [50–52]. Different countries around the world may design specific models based on their epidemiological situation or use a combination of several models. The models of UNICEF and WHO are among most important models and frameworks for prevention and control of malnutrition. In the UNICEF model consists of three levels of causes: basic, underlying, and immediate. Each of these levels contains components that need to be considered to prevent and control malnutrition [26]. The WHO model addresses the context and causes of malnutrition. Some other models, in addition to context causes, consider children and mothers as two main pillars, and necessitate a deeper understanding of cultural models in each region to formulate the needed programs [53, 54].

Policy options of the final model for malnutrition prevention in CU5 in Iran are presented based on educational, research, structural, economic, health-oriented, hygiene, political and inter-sectorial dimensions. This model was developed based on the current conditions in different regions of the country, factors related to child malnutrition, context effecting policy making, the content of previous policies, the process of policy making in Iran, and stakeholders and actors effective in policy making.

## Conclusion

The findings showed that in order to prevent malnutrition, first, the context causes should be identified and resolved. Context causes, solutions and interventions may differ in different regions of the country. The interventions can be supportive and health care related. The adopted policies should be strongly based on key stakeholders and actors. One of the most important needs in children nutrition, especially CU5, is formulating a comprehensive national document designed for this age group. The issue of targeted subsidies, helping poor people, job creation and production are also so important and need to be considered. Economic empowerment will ultimately lead to an increase in welfare of the family and the improvement of the nutritional status of children. One of the important strategies for improving economic conditions of the community is the balanced distribution of resources. Also, the correct reinforcing of laws will improve many indicators and ultimately improve the nutritional status of children. In many cases, there are proper and comprehensive rules for resolving issues but they are not correctly implemented. Finally, nutritional culture and literacy need to be considered. Low nutritional literacy will result in inadequate or inappropriate nutrition, malnutrition and other complications in children. Many of the current nutritional problems are due to wrong consumption culture in family that transfers to children. Solving the above-mentioned problems and meeting the desired goals require strong cooperation between the organizations involved in the nutrition of children; and it can be achieved with sufficient expertise and being commitment to the goals.

## Data Availability

The datasets used and/or analysed during the current study are available from the corresponding author on reasonable request.

## Abbreviations

CU5: Children under 5 years old
CMA: Comprehensive Meta-Analysis
UNICEF: The United Nations Children’s Fund
WHO: World Health Organization
STROBE: The Strengthening the Reporting of Observational studies in Epidemiology
